# North-to-south increasing gradient in *Toxoplasma gondii* seroprevalence and no serological evidence of exposure to *Neospora caninum* in semi-domesticated Eurasian tundra reindeer (*Rangifer tarandus tarandus*) in Finland and northern Norway in 2015

**DOI:** 10.1186/s13028-026-00869-2

**Published:** 2026-06-23

**Authors:** Pikka Jokelainen, Kayla J. Buhler, Eva Marie Breines, Morten Tryland, Sauli Laaksonen

**Affiliations:** 1 One Health and International Collaborations, Statens Serum Institut, Artillerivej 5, 2300 Copenhagen, Denmark; 2 Faculty of Veterinary Medicine, FINPAR, University of Helsinki, Agnes Sjöberginkatu 2, PL 66, 00014 Helsingin yliopisto, Helsinki, Finland; 3 Institute of Veterinary Medicine and Animal Sciences, Estonian University of Life Sciences, Kreutzwaldi 62, 51006 Tartu, Estonia; 4 Department of Forestry and Wildlife Management, Inland Norway University of Applied Sciences, Anne Evenstads Vei 8, 2480 Koppang, Norway; 5 Arctic Infection Biology, Department of Arctic and Marine Biology, UiT, The Arctic University of Norway, Framstredet 39, Breivika, N-9019 Tromsø, Norway

**Keywords:** Apicomplexa, Arctic, Epidemiology, Fennoscandia, Protozoa, Reindeer, Serology

## Abstract

**Background:**

While the coccidian parasites *Toxoplasma gondii* and *Neospora caninum* have a worldwide distribution and wide host ranges, relatively few studies have investigated these parasites in Eurasian tundra reindeer (*Rangifer tarandus tarandus*). The aim of this study was to estimate prevalence of antibodies against *T. gondii* and *N. caninum* in semi-domesticated Eurasian tundra reindeer in Finland and northern Norway.

**Results:**

Blood samples from 635 semi-domesticated reindeer were collected in 2015 and tested with commercial indirect enzyme-linked immunosorbent assays. Altogether 35 (5.5%; 95% confidence interval 3.9–7.5) of the reindeer were seropositive for *T. gondii*, while none of the reindeer tested seropositive for *N. caninum* (95% confidence interval 0.0-0.5). The seroprevalence for *T. gondii* was higher in adult animals (over 18 months) compared to calves, and there was a geographical north-to-south increasing gradient in seroprevalence.

**Conclusions:**

We detected serological evidence of exposure of semi-domesticated Eurasian tundra reindeer from Finland and northern Norway to the zoonotic parasite *T. gondii*, but not to *N. caninum*. The north-to-south increasing gradient in *T. gondii* seroprevalence may suggest that there is a gradient in environmental contamination with oocysts.

**Supplementary Information:**

The online version contains supplementary material available at https://doi.org/10.1186/s13028-026-00869-2.

## Background

Eurasian tundra reindeer (*Rangifer tarandus tarandus*) are found across northern Fennoscandia and parts of Russia. Apart from defined wild populations in Southern Norway, they are herded as semi-domesticated reindeer. They are free-ranging for most of the year on natural pastures, except for seasonal roundups and supplementary feeding. Reindeer pastoralism is practiced on large areas, e.g., on 36% of the land area of Finland, and it represents significant economic and cultural value for Sámi people [1].

*Toxoplasma gondii* is a zoonotic apicomplexan parasite of global importance that can infect a wide range of warm-blooded animals and humans [2]. The parasite has several infection routes and is maintained by chronically infected hosts as well as in the environment as sporulated, infective oocysts, which are originally shed in an unsporulated form in the faeces of infected felid definitive hosts [3, 4]. One of the transmission routes to humans is eating raw or undercooked meat of infected animals. Most infections in most host species are subclinical, but the parasite can also cause clinical disease manifestations in humans, in particular as a result of congenital infection and in immunocompromised individuals, as well as in animals – including in reindeer [2, 5]. The few reported *T. gondii* seroprevalence estimates in reindeer from Fennoscandia have been below 2% [6–9].

*Neospora caninum* is an apicomplexan parasite that causes abortions in cattle worldwide [10] and may have the ability to cause abortions and neurological disease in reindeer and caribou [11]. Canids are definitive hosts and can shed environmentally resistant oocysts in their faeces [12]. To our knowledge, there have been no reports of *N. caninum* in reindeer from Fennoscandia. However, seroprevalence estimate of 4.2% in reindeer kept in captivity in Germany [13] has been reported.

Both *T. gondii* and *N. caninum* can be transmitted in several ways, including vertically (transplacentally), by ingestion of tissue-dwelling forms of the parasites, and by ingestion of oocysts from the environment – the latter being considered the key transmission route for herbivorous hosts [14]. Detecting antibodies against these parasites in herbivores can serve as indicator for environmental oocyst contamination [15–17].

The main aim of this study was to estimate prevalence of antibodies against *T. gondii* and *N. caninum* in semi-domesticated Eurasian tundra reindeer in Finland and northern Norway. In addition, we investigated geographical origin and age as potential risk factors for seropositivity.

## Methods

### Ethical considerations

The reindeer included in this study were sampled at regular slaughter at slaughterhouses. The blood samples were collected after stunning, during bleeding. The reindeer were slaughtered for human consumption; no reindeer were slaughtered for the purpose of this study.

### Setting and study design

The study was designed as a cross-sectional seroepidemiological study investigating serological evidence of naturally-acquired *T. gondii* and *N. caninum* infections in reindeer. The target population was semi-domesticated Eurasian tundra reindeer in Fennoscandia, and a convenience multi-slaughterhouse study design was applied. The reindeer included in the study were apparently generally healthy, as they were evaluated to be suitable for slaughter for human consumption.

### Sampling

The samples were collected from September 30 to November 2, 2015, at nine reindeer slaughterhouses in Finland and one reindeer slaughterhouse in Norway (see map in [18]). Whole blood was collected directly into EDTA-tubes during bleeding of the animals. Upon arrival to the laboratory, the samples were centrifuged and the plasma stored frozen until the analyses.

### Serology

The samples were screened for specific anti-*T. gondii* antibodies using a commercial indirect ELISA (iELISA) kit (ID Screen^®^ Toxoplasmosis Indirect Multi-species, Idvet, Grabels, France). This iELISA kit has been used to investigate a wide range of animal species, including reindeer [9, 13]. Known negative and positive porcine serum samples provided in the kit were used as negative and positive controls, respectively.

The samples were tested for antibodies against *N. caninum* with a commercial iELISA (ID Screen^®^ Neospora caninum Indirect Multi-species, Idvet, Grabels, France). This iELISA kit has been used to investigate a range of animal species, including reindeer [13]. Negative and positive bovine control serum samples provided with the kit were used as negative and positive controls, respectively.

Samples were tested in single wells. Results were read as optical density (OD) and expressed relative to the positive control, as follows: (OD sample – OD negative control) / (OD positive control – OD negative control) x 100. The cutoff for interpretation as a positive was S/P% of 50 and the cutoff for a negative was S/P% of 40. Samples yielding results between the two cutoffs were considered doubtful and retested. Repeatedly doubtful serology results were considered as negative in the statistical analyses.

### Statistical analyses

Because sensitivity and specificity of the iELISA have not been validated in reindeer, we report apparent seroprevalence estimates. OpenEpi [19] was used to calculate the 95% confidence intervals (95% CI; Mid-P exact) and to evaluate the observed differences using two-by-two tables (two-tailed P-value; Mid-P exact). Logistic regression analyses were performed using Stata 13.1 (StataCorp, College Station, TX, USA). P-values < 0.05 were considered as significant. Correlation between the serology results for the two parasites (continuous variables) was evaluated visually and statistically. In addition to graphical methods, Shapiro-Wilk test was used to evaluate normality of these data.

The outcomes were dichotomised, meaning that each reindeer was either *T. gondii* seronegative or *T. gondii* seropositive, and either *N. caninum* seronegative or *N. caninum* seropositive, and the cut off for seropositivity was S/P% of 50 for both.

Age group and Region were evaluated as potential risk factors for testing seropositive. ‘Age group’ was a dichotomous variable, with each reindeer classified as either ‘calf’ (6–7 months) or ‘adult’ (over 18 months). Geographical Regions, each covering one to three reindeer herding cooperatives, were numbered so that Region 1 was the southernmost part of the study area and the Finnish reindeer herding area while Region 5 was the study area in Norway (see map in [18]). The ‘Regions’ were evaluated as five dummy variables with the southernmost Region 1 as the reference Region, and alternatively as continuous variable with the northernmost Regions 4 and 5 combined and coded with the lowest value. Univariable models were built first. Then, the two variables were offered to a model together and checked for presence of confounding. The performance metric area under the receiver operating characteristic (ROC) curve was used to express the predictive power of the models.

## Results

### Reindeer included in the sample

Samples from a total of 635 semi-domesticated reindeer were included in the study. The majority of the samples (*n* = 597; 94%) originated from northern Finland, from nine reindeer herding cooperatives, while a smaller proportion (*n* = 39; 6%) were from Finnmark County, the northern part of Norway. The samples comprised 384 samples from calves (60%) and 251 samples from adults (40%). The proportion of samples from adults was 46% in Region 3, 44% in Region 4, 40% in Region 2, 33% in Region 5, and 32% in Region 1.

### Serology results

**Fig. 1:**
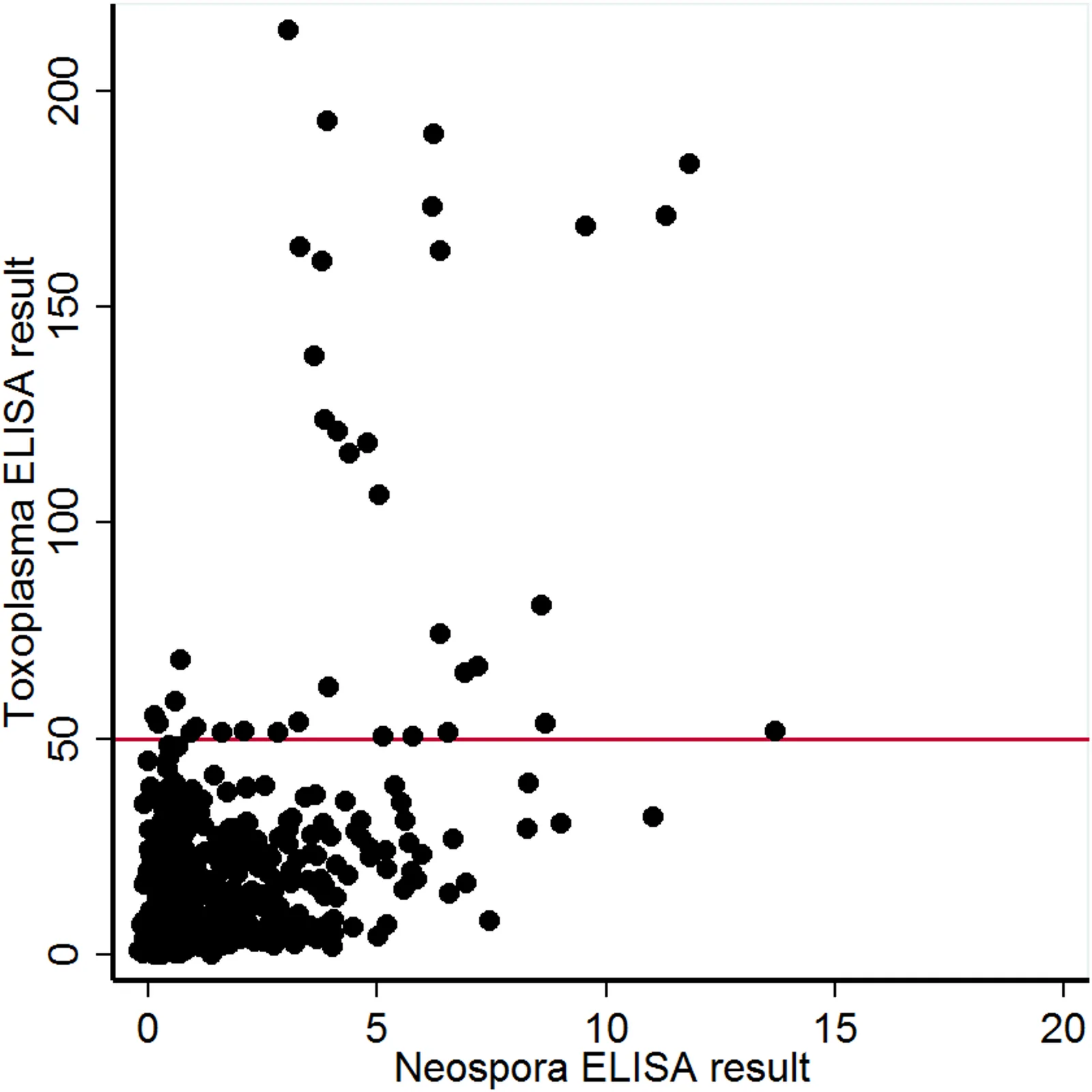
ELISA results for antibodies against *Toxoplasma gondii* and *Neospora caninum* in reindeer. The serology results of Eurasian tundra reindeer (*Rangifer tarandus tarandus*) tested with ELISAs for antibodies against *Toxoplasma gondii* (Y axis) and *Neospora caninum* (X axis). Results are expressed relative to positive control, as S/P%. The cutoff for seropositivity (S/P% of 50) is shown as a horizontal red line for *T. gondii*. The cutoff for *N. caninum* (S/P% of 50) lies outside the displayed range. Note the different scales of the axes

The *T. gondii* serology results (S/P%) ranged from − 0.06 to 214.21, mean was 16.89, and median was 8.89; the *N. caninum* serology results (S/P%) ranged from − 0.20 to 13.71, mean was 1.54, and median was 0.89. Both data differed significantly from a normal distribution. Spearman correlation coefficient was 0.400 (*P* < 0.001), indicating a weak to moderate positive correlation between the serology results for the two parasites, which are shown in Fig. 1.

### Toxoplasma gondii *seroprevalence*

Altogether 35 of the 635 reindeer were *T. gondii* seropositive, yielding an apparent seroprevalence of 5.51% (95% CI 3.93–7.50). Among the 600 samples interpreted as negative, there were six samples that tested repeatedly doubtful.

### Neospora caninum *seroprevalence*

None of the 635 reindeer were *N. caninum* seropositive, yielding an apparent seroprevalence of 0.00% (95% CI 0.00-0.47). There were no samples that were interpreted as negative due to testing doubtful twice.

### Risk factors for testing *T. gondii* seropositive

*Toxoplasma gondii* seroprevalence was significantly higher (*P* < 0.001) in adult reindeer than in calves (Table 1). Based on an univariable logistic regression model, adult reindeer had 4.14 (95% CI 1.95–8.77) times higher odds of being *T. gondii* seropositive than calves. The area under the ROC curve was 0.67, indicating this univariable model had moderate predictive power.

**Table 1:** *Toxoplasma gondii* seroprevalence in reindeer, by age group and region. *Toxoplasma gondii* seroprevalence in semi-domesticated Eurasian tundra reindeer (*Rangifer tarandus tarandus*) slaughtered in Finland and northern Norway in 2015, categorised by age group and geographical region. Region 1 was the southernmost part of the study area, and Region 5 was the study area in Finnmark, Norway. The seroprevalence estimates are presented with 95% confidence intervals

	*n* tested	*n* seropositive	% seropositive	95% confidence interval
Calf	384	10	2.60	1.33–4.59
Adult	251	25	9.96	6.70-14.14
Region 1	188	19	10.11	6.38–15.05
Region 2	129	10	7.75	4.01–13.38
Region 3	155	3	1.94	0.49–5.18
Region 4	124	2	1.61	0.27–5.23
Region 5	39	1	2.56	0.13-12.00
Total	635	35	5.51	3.93–7.50

Based on an univariable logistic regression model using the Regions as dummy variables, being from Region 3 and being from Region 4 appeared as statistically significant protective factors against being *T. gondii* seropositive, with odds ratios of 0.18 (95% CI 0.05–0.60) and 0.15 (95% CI 0.03–0.64), respectively. The area under the ROC curve was 0.69, indicating moderate predictive power.

A north-to-south increasing gradient in *T. gondii* seroprevalence was apparent (Table 1). *Toxoplasma gondii* seroprevalence was highest (over 10%) in the southernmost Region 1 (Table 1) and lower in the more northern Regions. An univariable model using the combined northernmost Region and the other Regions as continuous variable suggested that the odds of being *T. gondii* seropositive would almost double (increase with a factor of 1.94, 95% CI 1.36–2.75) from one Region to the next from north to south. When the variable ‘Age group’ was added to this model, the factor became 2.14 (95% CI 1.48–3.10), while adults had 5.08 (95% CI 2.35–10.98) times higher odds to test *T. gondii* seropositive than calves. This model had good predictive power with the area under the ROC curve being 0.76.

Based on the final logistic regression model (Table 2), adult reindeer had higher odds of being *T. gondii* seropositive than calves, and reindeer from Regions 3 and 4 had lower odds of being *T. gondii* seropositive than reindeer from Region 1. The odds ratios differed from those from the univariable analysis, indicating confounding. The final model had good predictive power with the area under the ROC curve being 0.76.

**Table 2:**
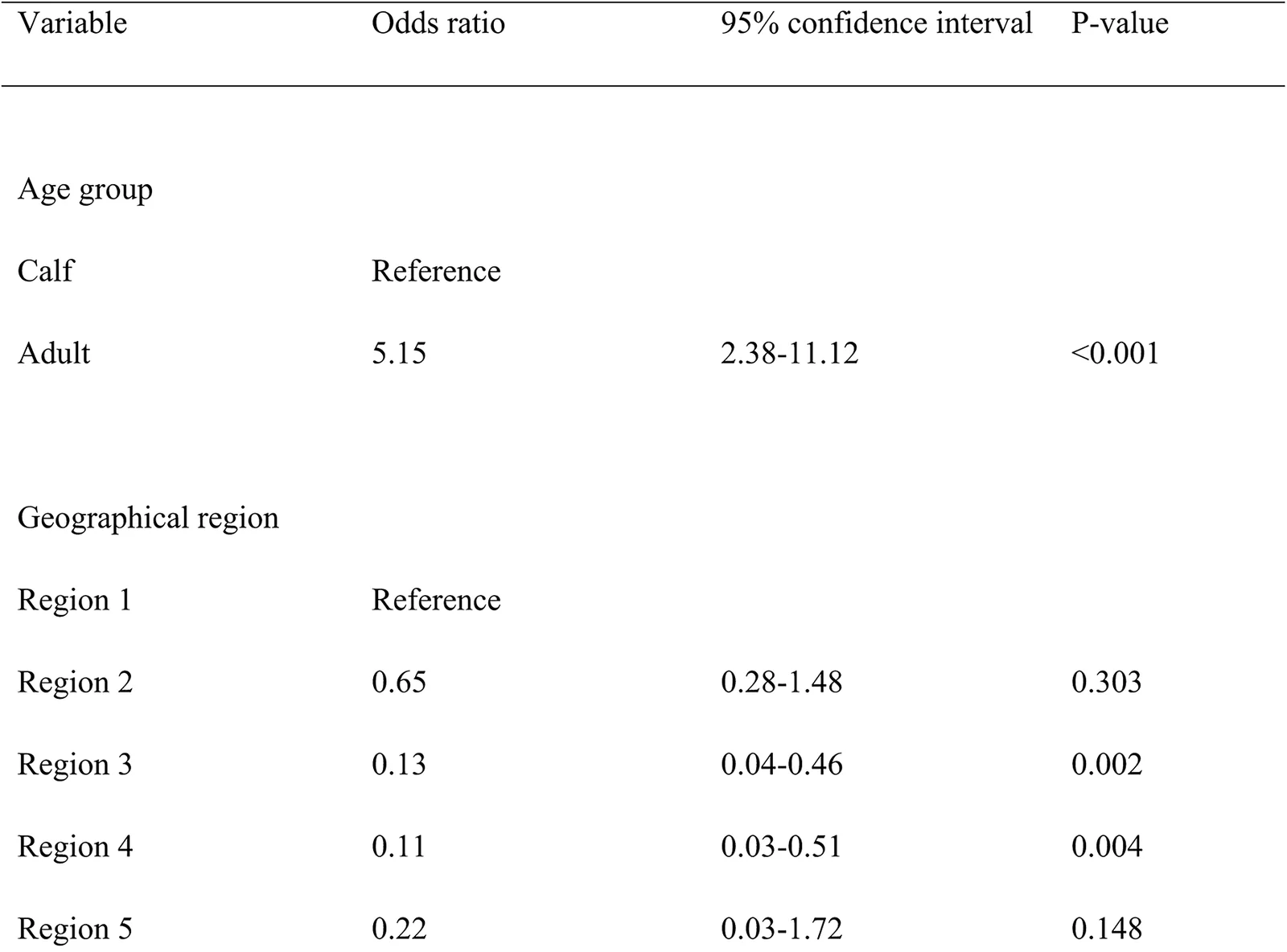
Logistic regression model for *Toxoplasma gondii* seropositivity in reindeer. The final logistic regression model for testing *Toxoplasma gondii* seropositive, with age group and geographical region as predictors, estimated using data from 635 semi-domesticated Eurasian tundra reindeer (*Rangifer tarandus tarandus*) slaughtered in Finland and northern Norway in 2015. Calves and the southernmost Region 1 served as reference categories. Results are shown as odds ratios with 95% confidence intervals and associated P-values

## Discussion

The results of this work add to the limited literature available on the zoonotic parasite *T. gondii* in reindeer. Our study identified a higher overall seroprevalence (5.5%) when compared to a previous study that was based on samples from Finland and relatively few samples from Finnmark county, Norway, which reported that 0.9% (23/2577) of reindeer had antibodies for *T. gondii* [7]. The difference in seroprevalence between time frames may be influenced by multiple factors, including different age distribution among the reindeer convenience sampled, different serological tests used (direct agglutination test versus iELISA), or differences in exposure due to changing environmental factors and herding practices. The management of reindeer herds has developed towards more intensive herding and supplementary feeding in corrals during winter months [20]. Given that *T. gondii* seropositivity has been positively associated with degree of domestication and corral feeding [7], the higher seroprevalence might reflect herd management changes. Further studies should monitor changes in exposure to these parasites, because changes in herd management practices as well as climate and environment changes may provide more opportunities for survival and transmission of these parasites.

Reported estimates of *T. gondii* seroprevalence in free-ranging caribou populations from Alaska (0.4%; 2/452) and Canada (1.8%; 13/706) have been lower than the estimates from our study [21, 22]. In addition, our study reports a higher seroprevalence in both adults (9.96%) and calves (2.6%) when compared to estimates from the neighbouring country Sweden, where 2.3% (5/209) and 1.9% (6/308) of adults and calves, respectively, had antibodies against *T. gondii* [9]. By contrast, a substantially higher proportion, 52.1% (62/119), of reindeer kept in captivity in Germany tested positive [13]. Comparison with the Swedish and German studies is interesting as the samples were tested with the same commercial kit, and the samples in the neighbouring country Sweden were collected a year prior to those included in our study.

Being an adult was a risk factor for *T. gondii* seropositivity in our study, and one tenth of the adult reindeer were positive for antibodies. This is in line with observations from many other animal species, in which most *T. gondii* infections are chronic and subclinical, as older animals have had a longer time to encounter the parasite [23]. It is important to note that our samples did not include very young calves; the overall seroprevalence estimate can be an overestimation, because among very young calves, a relatively low seroprevalence would be expected, and inclusion of the age group would be expected to lower the overall seroprevalence. As experimental infections have revealed that toxoplasmosis can be fatal in reindeer [24], it cannot be ruled out that our results could be affected by possible mortality caused by the parasite; the seroprevalence estimate might be an underestimation, because any exposed individuals that died from the infection would not be included in the sample.

The geographical differences observed in *T. gondii* seroprevalence were not explained by the different age distribution of the animals. In contrast, the proportion of adults were highest in reindeer from the two Regions (3 and 4) that appeared as significant protective factors against *T. gondii* seropositivity when compared with Region 1. Increasing north-to-south gradient in the seroprevalence was evident, similar to earlier observations in free-ranging moose (*Alces alces*) across Finland [15]. Gradient of *T. gondii* exposure has been observed also earlier in semi-domesticated reindeer in Finland and Finnmark (Norway), and it has been suggested to be linked to corral feeding [7]. However, the gradient could also reflect the degree of environmental contamination with *T. gondii* oocysts, as reindeer are mainly herbivorous and likely to acquire the infection from oocysts. The presence of seropositive reindeer indicates exposure to the zoonotic parasite, likely from the environment, which is shared by other animals and humans.

Degree of domestication and environmental contamination with oocysts are probably not independent of each other, given that the most likely source of oocysts are domestic cats. Infected felids can shed the oocysts of *T. gondii*, and the population of humans and their domestic cats, as well as the population of Eurasian lynx (*Lynx lynx*), are concentrated in the southern parts of Finland. The parasite is common in domestic cats in the country, with seroprevalence estimates ranging from 35.6% in shelter cats to 49.7% in purebred pet cats, with variation by cat breed [25, 26], and seroprevalence in wild free-ranging Eurasian lynx is high, 86.1% [27]. While the sporulated oocysts are robust against environmental factors, different environmental conditions can affect their survival in the environment. If climatic conditions partly explain the north-to-south gradient in *T. gondii* seroprevalence, a warming climate might mean increased exposure in the future.

None of the reindeer included in this study tested positive for *N. caninum* antibodies. This result is in contrast to the seroprevalence estimates reported for reindeer kept in captivity in Germany (4.2%, 5/118) [13] and free-ranging caribou from Alaska (11.5%; 45/390) and Canada (26.7%; 131/490) [21, 22]. It is important to note that different serological methods were used in these studies; the method used in Germany was the same as the one used in this study. However, the relatively high seroprevalence in North America might be due to differences in the density of hosts that can shed *N. caninum* oocysts in the environment. The northern latitudes of North America are home to multiple wild hosts for *N. caninum*, including gray wolves (*Canis lupus*) and coyotes (*C. latrans*) [28]. The number of wild canid species and their population in Fennoscandia are lower [29, 30]. Domestic dogs, if infected, are also possible sources of *N. caninum* oocysts. Different subpopulations of domestic dogs, if fed raw meat, may contribute to the risk for semi-domesticated reindeer. Dogs are kept in the area as pets as well as for working, e.g., for hunting, as sledge dogs, and as herding dogs.

Seroprevalence results should be interpreted with some caution. Multi-host-species kits, like the iELISAs used in this study, are useful for testing for pathogens with wide host ranges. However, challenges include the lack of species-specific control sera and validated cut-off values as well as sensitivity and specificity for different host species, and additionally the lack of suitable confirmatory tests. Testing the same samples for several pathogens, as done in this work, provides important information about performance of the tests. The absence of dual seropositivity suggests that major cross-reactivity is unlikely in this dataset. There was a weak to moderate statistical correlation between the serology results for the two pathogens, but it should be emphasised that the scale was ten-fold higher for *T. gondii* results and that none of the reindeer were interpreted as *N. caninum* seropositive (Fig. 1).

Another limitation in our study is the bias that can be introduced when sampling at slaughterhouses, e.g., excluding animals that are kept for breeding purposes and animals that have died in the field. Inclusion of multiple slaughterhouses, on the other hand, increased representativeness. There is no known biological mechanism by which *T. gondii* or *N. caninum* serostatus would systematically influence whether a semi-domesticated reindeer is sent to slaughter during the normal slaughter season. Both infections are typically subclinical in naturally infected individuals of most host species, and seropositivity reflects prior exposure rather than acute disease.

Sampling reindeer at slaughterhouses is relevant from food safety and One Health perspectives, as the reindeer included in our study were slaughtered for human consumption. Generally, eating undercooked meat of infected animals is a known risk factor for human *T. gondii* infections [31, 32]. Reindeer meat inspection at slaughterhouses does not attempt to detect *T. gondii* [33]. While the methodology used in our study was indirect (antibody detection) and did not directly show the presence of infective parasites, the results show that reindeer in Finland and northern Norway are exposed to the zoonotic parasite *T. gondii*. Cooking meat thoroughly eliminates the possibility of meatborne transmission, as tissue cysts are destroyed by heating to 67 °C [34].

## Conclusions

We detected serological evidence of exposure to *T. gondii*, but not to *N. caninum*, in semi-domesticated Eurasian tundra reindeer from Finland and northern Norway. There was an increase from previous overall estimates, possibly reflecting changes in herd management, and a north-to-south increasing gradient in *T. gondii* seroprevalence, suggesting that there is a gradient in environmental contamination with *T. gondii* oocysts. Further studies should monitor changes in the prevalences, as herd management practices and climate and environment changes may provide more opportunities for transmission of these parasites.

## Supplementary Information

Supplementary Material 1. The serology results of each individual reindeer. Dataset containing the serology results for antibodies against *Toxoplasma gondii* and *Neospora caninum* of each of the 635 semi-domesticated Eurasian tundra reindeer (*Rangifer tarandus tarandus*) sampled in Finland and northern Norway in 2015.

## Data Availability

The dataset is provided as Additional file 1.
